# No survival benefit could be obtained from adjuvant radiotherapy in esophageal cancer treated with neoadjuvant chemotherapy followed by surgery: A SEER-based analysis

**DOI:** 10.3389/fonc.2022.897476

**Published:** 2022-09-14

**Authors:** Si-Yue Zheng, Wei-Xiang Qi, Sheng-Guang Zhao, Jia-Yi Chen

**Affiliations:** Department of Radiation Oncology, Ruijin Hospital, Shanghai Jiaotong University School of Medicine, Shanghai, China

**Keywords:** esophageal cancer, postoperative radiotherapy, neoadjuvant chemotherapy, SEER, prognosis

## Abstract

**Background:**

The aim of this study is to assess the clinical benefit of postoperative radiotherapy (PORT) in patients with esophageal cancer (EC) who treated with neoadjuvant chemotherapy (NAC) and surgery *via* a national population-based database.

**Methods:**

Patients diagnosed with EC between 2004 and 2015 were identified from the Surveillance, Epidemiology, and End Results (SEER) database. Kaplan–Meier survival analysis was used to compare the overall survival (OS) and cause-specific survival (CSS) difference between PORT *vs*. no-radiotherapy (RT) groups before and after propensity score matching (PSM). After PSM for baseline characteristics, Cox proportional hazard regression was performed to investigate the factors associated with OS.

**Results:**

A total of 321 patients were included in the analysis. Of them, 91 patients (28%) received PORT. In the unmatched population, the no-RT group had improved OS compared with PORT (44 *vs*. 25 months, p = 0.002), and CSS was similar in patients undergoing NAC with or without PORT (42 *vs*. 71 months, p = 0.17). After PSM for baseline characteristics, the OS benefit of the no-RT group over the PORT group remained significant with a median OS of 46 *vs*. 27 months (p = 0.02), and CSS remained comparable between groups (83 *vs*. 81 months, p = 0.49). In subgroup analyses, PORT did not improve the OS among patients with adenocarcinoma in the subgroups of cN0, cN1, and cN2–3 (all p > 0.05). In Cox regression, aged ≥71 years old, cT3–4, cN2–3, and receiving PORT were independent predictors of worse OS, whereas cT4 and cN2–3 were independent predictors of worse CSS (all p < 0.05).

**Conclusions:**

The present study demonstrated that no survival benefit could be obtained from the additional use of PORT after NAC and surgery in patients with EC. Well-designed prospective trials are needed to confirm our findings.

## Introduction

In 2020, around 1 in every 18 cancer deaths is attributed to esophageal cancer (EC), which is now the seventh most common cancer and ranks sixth in mortality worldwide (1). Although esophagectomy is generally accepted as the mainstay treatment for decades, neoadjuvant and adjuvant therapies have been performed to improve the overall survival (OS) among these patients. The benefits of neoadjuvant chemotherapy (NAC) in EC have primarily been proven in the MAGIC trial, in which perioperative chemotherapy was superior to surgery alone for patients with gastroesophageal adenocarcinoma in terms of OS and progression-free survival (2). As demonstrated by the CROSS trial, neoadjuvant chemoradiotherapy (nCRT) could significantly prolong OS and disease-free survival (DFS) in patients with locally advanced EC compared with surgery alone (3). Subsequently, the NEOCRTEC 5010 trial also confirmed that treatment with nCRT plus surgery significantly improved long-term OS and DFS for patients with locally advanced esophageal squamous cell carcinoma (ESCC) (4).

However, the superiority of nCRT over NAC alone has not been evaluated in EC. Although few randomized controlled trials of small sample and meta-analyses have been performed to compare these two treatment modalities, controversy existed because of inconsistent conclusions and limited sample size (5–8). On the other hand, many patients with EC with poor performance status, older age, or comorbidities may be ineligible for nCRT due to expected high toxicity (9).

Meanwhile, the optimal postoperative therapeutic strategy remains undetermined. For those patients who undergo surgery without neoadjuvant therapy, several studies investigated the role of postoperative radiotherapy (PORT) in EC but reached conflicting conclusions (10–13). A prospective randomized study of 495 patients shows that PORT could improve the 5-year survival in patients with EC with positive lymph nodes and those with stage III disease (10). On the other hand, another prospective randomized study of 68 patients found no significant difference between the surgery alone group and the PORT group, and PORT significantly increased the incidence of esophagogastric fibrosis and affected the quality of life (11).

To date, the benefit of PORT in patients with EC undergoing NAC and surgery is not well established. Therefore, we sought to compare the survival benefit of patients with EC treated with and without PORT following NAC and surgery.

## Material and methods

### Patients

**Figure 1:**
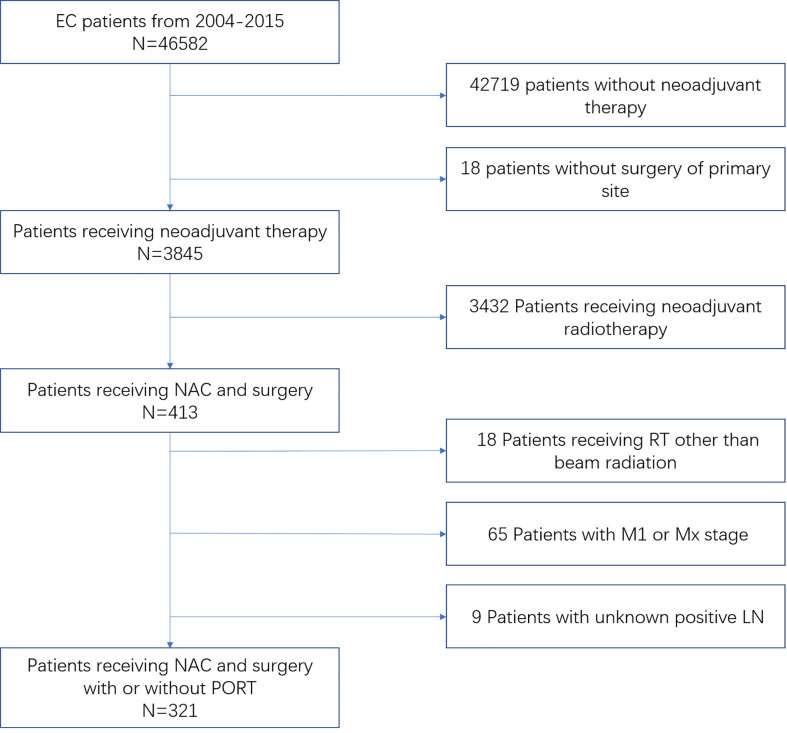
Inclusion and exclusion flow diagram for SEER EC patients receiving NAC followed by surgery with or without PORT from 2004 to 2015.

This population-based study was performed by using data from the Surveillance, Epidemiology, and End Results (SEER) database to identify patients with EC who underwent NAC and surgery diagnosed from 2004 to 2015. We obtained permission to access SEER Research Plus Data, Nov 2019 Sub (1975–2017) with reference number 11564-Nov2019. Cases eligible were required to have confirmed diagnosis with the recode as “only malignant in ICD-O-3” and the primary tumor site of the esophagus. Patients who received preoperative therapy without radiation prior to surgery were considered as having received NAC and included for analysis. For the sequence and type of radiation, only external beam radiation after surgery or no radiation was included for analysis. The following covariates were included: year of diagnosis, age, gender, race, chemotherapy, radiotherapy (RT) type and sequence, tumor histology, histological grade, clinical tumor (cT) stage, clinical nodal (cN) stage, clinical metastasis (cM) stage, and vital status, which includes the cause of death and the follow-up duration. cT, cN, and cM stages were categorized on the basis of the sixth edition of the American Joint Committee on Cancer/Union for International Cancer Control staging guidelines, and only cM0-stage patients were eligible. Patients with inadequate information were excluded from the final analysis. A flow diagram for patient inclusion and exclusion is shown in **Figure 1**.

### Statistical analysis

The chi-square test was used to compare the differences for categorical variables in clinicopathologic features between RT and no-RT groups. A propensity score matching (PSM) analysis (1:2 ratio; method, nearest neighbor matching; caliper, 0.03) was performed to balance the observed characteristics between the two groups. OS and cause-specific survival (CSS) were estimated by the Kaplan–Meier method, and the log-rank test was applied to compare survival curves. Univariate and multivariate Cox regression models were performed to investigate risk factors for OS. The variables with p ≤ 0.10 in the univariate model were subsequently included in the multivariate analysis. All statistical analyses were performed in IBM SPSS version 23.0 and R statistical software version 4.0.3. Two-sided p < 0.05 was considered as statistically significant.

## Results

### Patient characteristics

From 2004 to 2015, a total of 321 patients registered in the SEER database received NAC alone followed by esophagectomy; the mean age at diagnosis was 62.41 ± 8.99 years. Of these, 230 patients (72%) did not receive adjuvant external beam radiation after surgery, whereas 91 patients (28%) received PORT.

The majority of the patients were of age from 61 to 70 years (43.9%), white (89.7%), and men (86.2%). The most frequent histological type was adenocarcinoma at 81.3% followed by ESCC at 18.7%. Notably, the patients who were treated with PORT tended to have a higher cN classification and a worse differentiated histological grade, whereas there was no statistically significant difference between the two groups in terms of age, sex, race, year of diagnosis, tumor histology, and cT classification. With PSM consisting of the number of positive lymph nodes and histological grade, 79 patients treated with PORT were successfully matched with 140 patients who did not receive postoperative radiation. The baseline clinicopathological characteristics for the study population before and after PSM are demonstrated in **Table 1**.

**Table 1:** Baseline characteristics of patients included in the analysis before and after PSM.

Characteristic	Before PSM			After PSM		
	Without PORT (n, %)	With PORT (n, %)	P-value	Without PORT (n, %)	With PORT (n, %)	P-value
**Total**	n = 230	n = 91		n = 140	n = 79	
**Year of diagnosis**			0.337			0.728
2004–2007	60 (26.1)	31 (34.1)		43 (30.7)	26 (32.9)	
2008–2011	101 (43.9)	34 (37.4)		59 (42.1)	29 (36.7)	
2012–2015	69 (30.0)	26 (28.6)		38 (27.1)	24 (30.4)	
**Gender**			0.211			0.07
Male	195 (84.8)	82 (90.1)		115 (82.1)	72 (91.1)	
Female	35 (15.2)	9 (9,9)		25 (17.9)	7 (8.9)	
**Age groups (years)**			0.838			0.911
≤50	19 (8.3)	10 (11.0)		12 (8.6)	8 (10.1)	
51–60	68 (29.6)	27 (29.7)		38 (27.1)	23 (29.1)	
61–70	101 (43.9)	40 (44.0)		64 (45.7)	36 (45.6)	
≥71	42 (18.3)	14 (15.4)		26 (18.6)	12 (15.2)	
**Race**			0.337			0.365
White	204 (88.7)	84 (92.3)		124 (88.6)	73 (92.4)	
Black and others	26 (11.3)	7 (7.7)		16 (11.4)	6 (7.6)	
**cT classification**			0.862			0.606
T1	34 (14.8)	12 (13.2)		18 (12.9)	12 (15.2)	
T2	37 (16.1)	15 (16.5)		22 (15.7)	15 (19.0)	
T3	141 (61.3)	59 (64.8)		89 (63.6)	49 (62.0)	
T4 and Tx	18 (7.8)	5 (5.5)		11 (7.9)	3 (3.8)	
**cN classification**			0.004			0.832
N0	134 (58.3)	36 (39.6)		66 (47.1)	35 (44.3)	
N1	54 (23.5)	24 (26.4)		46 (32.9)	24 (30.4)	
N2	26 (11.3)	23 (25.3)		23 (16.4)	16 (20.3)	
N3	16 (7.0)	8 (8.8)		5 (3.6)	4 (5.1)	
**Tumor histology**			0.448			0.896
Adenocarcinoma	195 (84.8)	74 (81.3)		116 (82.9)	66 (83.5)	
SCC	35 (15.2)	17 (18.7)		24 (17.1)	13 (16.5)	
**Histological grade**			0.041			0.865
Well	6 (2.6)	7 (7.7)		4 (2.9)	4 (5.1)	
Moderate	87 (37.8)	25 (27.5)		43 (30.7)	24 (30.4)	
Poor/Undifferentiated	113 (49.1)	53 (58.2)		83 (59.3)	46 (58.2)	
Unknown	24 (10.4)	6 (6.6)		10 (7.1)	5 (6.3)	

PSM, propensity score matching; PORT, postoperative radiotherapy; T, tumor; N, nodal; SCC, squamous cell carcinoma.

### Survival prior to PSM

**Figure 2:**
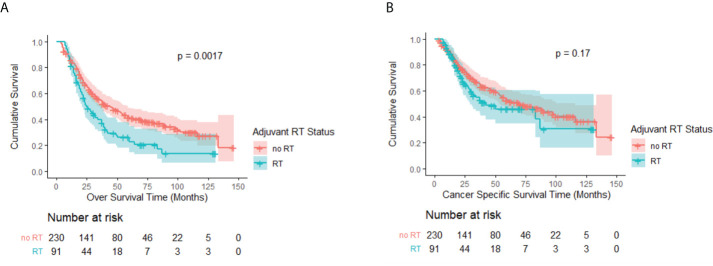
**(A)** Kaplan–Meier OS curve by adjuvant RT status before PSM. A significant difference was noted (P = 0.002). **(B)** Kaplan–Meier CSS curve by adjuvant RT status before PSM. No significant difference was observed (P = 0.17).

The median follow-up time for the eligible patients was 74 months [interquartile range (IQR), 47–109 months] with the median OS being 37 months (IQR, 18–116 months). **Figures 2A, B** represent a Kaplan–Meier OS curve and a Kaplan–Meier CSS curve with the number of subjects at risk and 95% confidence interval (CI) comparing patients who either received or did not receive PORT. The results of the log-rank test are also shown in **Figures 2A, B**. A significant OS benefit was noted between the no-RT and RT groups (P = 0.002). The median OS rates for patients who received and did not receive PORT were 25 months (95% CI, 18.7–31.3 months) and 44 months (95% CI, 32.6–55.4 months), respectively. The log-rank test did not indicate a significant CSS difference between the two groups (P = 0.17). However, the patients not receiving PORT still had a longer median CSS of 71 months (95% CI, 46.7–95.3 months), followed by the patients receiving RT only with a CSS of 42 months (95% CI, 7.6–76.4 months).

### Survival after PSM

**Figure 3:**
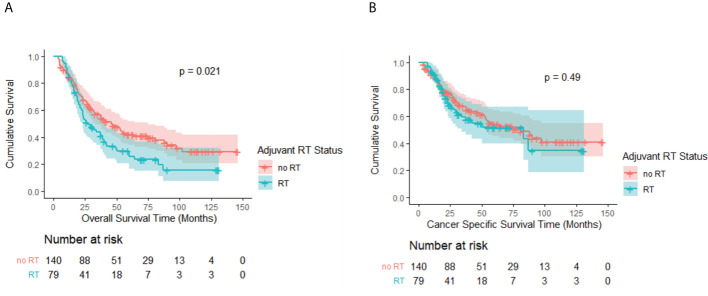
**(A)** Kaplan–Meier OS curve by adjuvant RT status after PSM. A significant difference was noted (P = 0.02). **(B)** Kaplan–Meier CSS curve by adjuvant RT status after PSM. No significant difference was observed (P = 0.49).

In the matched cohort, the OS advantage of the no-RT group over the RT group persisted with a median OS of 46 months (95% CI, 33.3–58.7 months) and 27 months (95% CI, 16.9–37.1 months), respectively (p = 0.02; **Figure 3A**). CSS remained comparable between the groups (p = 0.49; **Figure 3B**). The no-RT group still had more favorable median CSS of 83 months (95% CI, 49.2–112.8 months) versus 81 months (95% CI, 22.2–143.8 months) for the RT group.

**Figure 4:**
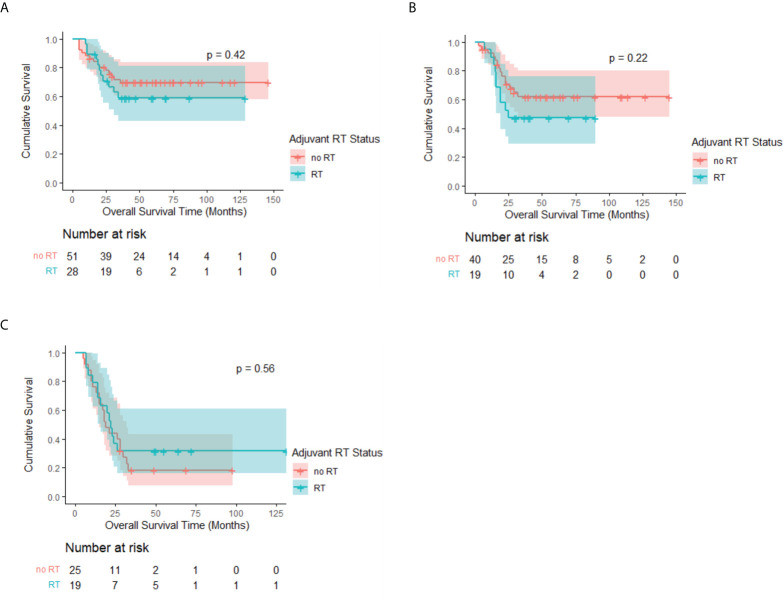
Kaplan–Meier OS curve of patients with adenocarcinoma by adjuvant RT status after PSM, stratified by cN stage. Median survival estimates: **(A)** cN0: Both median OS not reached, p = 0.42. **(B)** cN1: Both median OS not reached, p = 0.22. **(C)** cN2–3: RT: 22 months (95% CI, 17.7–26.3 months) *vs*. No RT: 19 months (95% CI, 14.1–23.9 months), p = 0.56.

Moreover, Kaplan–Meier analysis stratified by the cN stage among patients with adenocarcinoma revealed no statistical significance between the RT and no-RT groups. The median OS rates of the two groups were all not reached in the cN0 and cN1 subgroups, whereas the 3-year OS rates of the RT group were higher than those of the no-RT group but showed no significance (cN0: 69.7% *vs*. 58.9%, p = 0.42, **Figure 4A**; cN1: 61.9% *vs*. 47.4%, p = 0.22, **Figure 4B**). There is also no survival benefit of PORT in the cN2–3 subgroup (median OS: 22 months *vs*. 19 months, p = 0.56, **Figure 4C**).

The prognostic factors associated with OS in univariate and multivariate analyses for the matched cohort are shown in **Table 2**. Univariate analysis showed that the factors associated with worse OS included age ≥71 years old, cT3–4, cN2–3, and receiving PORT, which remained independent factors significantly decreasing OS in multivariate analysis.

**Table 2:** Univariate and multivariate analysis of OS for the matched cohort after PSM.

Characteristic	Univariate			Multivariate		
	P-value	OR	95% CI	P-value	OR	95% CI
**Year of diagnosis**	0.599					
2004–2007	1					
2009–2011	0.731	0.936	0.641–1.367			
2012–2015	0.314	0.783	0.487–1.260			
**Gender**						
Male	1					
Female	0.135	0.671	0.397–1.132			
**Age groups (years)**	0.024			0.002		
≤50	1			1		
51–60	0.976	1.01	0.521–1.956	0.511	1.262	0.630–2.526
61–70	0.381	1.318	0.711–2.444	0.068	1.817	0.957–3.451
≥71	0.032	2.098	1.068–4.123	0.002	3.052	1.496–6.228
**Race**						
White	1					
Black and others	0.293	0.708	0.372–1.348			
**cT classification**	0.004			0.003		
T1	1			1		
T2	0.45	1.306	0.654–2.609	0.399	1.358	0.667–2.763
T3	0.01	2.153	1.205–3.847	0.013	2.132	1.177–3.862
T4	0.002	4.236	1.684–10.652	0.001	4.91	1.888–12.768
Tx	0.852	0.868	0.196–3.846	0.745	0.777	0.170–3.546
**cN classification**	< 0.001			< 0.001		
N0	1			1		
N1	0.158	1.332	0.895–1.984	0.159	1.337	0.892–2.002
N2	0.001	2.106	1.349–3.288	0.001	2.177	1.359–3.486
N3	< 0.001	4.597	2.237–9.449	<0.001	4.079	1.965–8.467
**Tumor histology**						
Adenocarcinoma	1					
SCC	0.934	1.019	0.651–1.596			
**Histological grade**	0.567					
Well	1					
Moderate	0.251	1.985	0.616–6.393			
Poor/Undifferentiated	0.285	1.876	0.592–5.944			
Unknown	0.169	2.453	0.684–8.800			
**PORT**						
Yes	1			1		
No	0.024	0.674	0.479–0.948	0.012	0.638	0.448–0.907

T, tumor; N, nodal; SCC, squamous cell carcinoma; PORT, postoperative radiotherapy.

The prognostic factors associated with CSS in univariate and multivariate analyses for the matched cohort are shown in **Table 3**. On univariate analysis, the factors associated with worse CSS included male sex, cT3–4, cN2–3, and adenocarcinoma. On multivariate analysis, cT4 and cN2–3 were still independently associated with a decreased CSS.

**Table 3:** Univariate and multivariate analysis of CSS for the matched cohort after PSM.

Characteristic	Univariate			Multivariate		
	P-value	OR	95% CI	P-value	OR	95% CI
**Year of diagnosis**	0.903					
2004–2007	1					
2009–2011	0.838	0.952	0.592–1.530			
2012–2015	0.651	0.875	0.490–1.562			
**Gender**						
Male	1			1		
Female	0.056	0.491	0.237–1.018	0.148	0.574	0.270–1.219
**Age groups (years)**	0.554					
≤50	1					
51–60	0.617	1.223	0.556–2.694			
61–70	0.657	1.188	0.555–2.546			
≥71	0.215	1.715	0.732–4.021			
**Race**						
White	1					
Black and others	0.177	0.537	0.218–1.325			
**cT classification**	0.001			0.002		
T1	1			1		
T2	0.650	0.807	0.320–2.034	0.454	0.697	0.270–1.797
T3	0.034	2.127	1.058–4.276	0.083	1.880	0.921–3.838
T4	0.002	5.201	1.843–14.676	0.009	4.107	1.420–11.875
Tx	0.647	0.617	0.078–4.874	0.423	0.424	0.052–3.455
**cN classification**	0.001			0.004		
N0	1			1		
N1	0.176	1.407	0.857–2.310	0.269	1.330	0.802–2.206
N2	0.003	2.285	1.318–3.962	0.010	2.116	1.195–3.748
N3	< 0.001	4.859	2.003–11.789	0.002	4.134	1.688–10.127
**Tumor histology**						
Adenocarcinoma	1			1		
SCC	0.031	0.428	0.198–0.926	0.129	0.538	0.242–1.198
**Histological grade**	0.410					
Well	1					
Moderate	0.380	1.902	0.453–7.989			
Poor/Undifferentiated	0.406	1.821	0.443–7.487			
Unknown	0.156	3.037	0.655–14.071			
**PORT**						
Yes	1					
No	0.494	1.164	0.754–1.797			

T, tumor; N, nodal; SCC, squamous cell carcinoma; PORT, postoperative radiotherapy.

## Discussion

According to the National Comprehensive Cancer Network guideline (version 3.2021) recommendation, all patients with EC who have not received nCRT or NAC with R1 or R2 resection should receive PORT. For R0 cases, PORT is only recommended for T3–T4a or N1–3 patients with adenocarcinoma without nCRT or NAC (14). However, the efficacy of adding PORT in patients with EC after NAC alone remains unclear. To the best of our knowledge, this is the first retrospective study to investigate the role of PORT for patients with EC after NAC and surgery.

We revealed that omitting PORT after NAC and surgery showed a significantly better OS than the PORT group before and after PSM, whereas there were no significant differences in CSS between the two groups. In subgroup analysis according to recurrence risk factors, we also found that no survival benefit could be obtained in those with cT3 stage or positive nodes, which was quite different from previous studies focusing on the effectiveness of PORT in patients with EC without defining the use of NAC (15, 16). This may be attributed to the treatment toxicities caused by PORT, which have already been affected by the chemotherapy and surgery. Wang et al. revealed that 18% of the patients with EC experienced grade 3 or higher cardiac events after RT, which was associated with worse OS (p = 0.041) (17). Pinder-Arabpour et al. demonstrated that ventilation heterogeneities occurred in 30% of the patients with EC undergoing RT (18). Although currently we did not find any research comparing the side effects between NAC + surgery with and without PORT, Zhang et al. reported that NAC caused fewer cardiopulmonary events than nCRT (19). The patients analyzed in our study were diagnosed in the years from 2004 to 2015, and most of them received conventional radiation therapy by using two parallel beams with opposed orientations. Therefore, relatively large volumes of normal tissues adjacent to the treatment field (including the mediastinum, chest wall, and adjacent lung) are irradiated. Further investigations with advanced technology such as intensity-modulated radiation therapy and proton therapy are in progress to confirm the safety of the treatment strategy (NCT01512589).

In our study, adenocarcinoma accounts for 83.8% of all 321 patients, which reflects the high prevalence of adenocarcinoma in Western countries just as most clinical trials conducted in Europe and Northern America (2, 3, 20). Conversely, considering that SCC was the most common histological subtype among Chinese patients with EC, the conclusion might not be directly applied to East Asia people (21). As the 10-year outcome of the CROSS trial demonstrated, nCRT tended to be more beneficial in the SCC group than in the adenocarcinoma group with a 10-year OS in the nCRT-surgery group of 46% and 36%, respectively (22). The conclusion was confirmed by the NEOCRTEC5010 trial, in which the median OS for Chinese patients with ESCC receiving nCRT plus surgery was 100.1 months and the 3-year OS was 69.1%, which is obviously better than that reported in previous trials containing more patients with adenocarcinoma (4, 20). On the other hand, NAC is suggested as the standard treatment for locally advanced ESCC in Japan according to the result of the JCOG9907 trial, in which the 5-year OS of the NAC group was 55% (23, 24). In our study, the 3- and 5-year OS rates of patients with SCC in the PORT group after PSM were 25.6% and 17.1%, respectively, and those in the no-RT group were 56.1% and 44.9%, respectively. Comparing our results with those of the clinical trials mentioned above, the OS of both groups in our study showed a reduction by at least 10% compared with the prognosis in the NEOCRTEC5010 and JCOG9907 trials. Taken together, the present study demonstrated that the addition of PORT to NAC combined with surgery in patients with ESCC may also be associated with a higher mortality and adjuvant RT is also not recommended in patients with ESCC treated with NAC.

It is also worth mentioning that immunotherapy has shown positive impacts on patients with advanced EC from back line to first line, according to the result of several clinical trials such as KEYNOTE-590 and ESCORT-1st, but little is confirmed about its role in neoadjuvant therapy regimen (25, 26). Some single-armed trials focused on preoperative immuno-chemo-radiotherapy. For example, the PERFECT trial combined Atezolizumab with nCRT, and the pathologic complete response (PCR) rate was 25% (27). PALACE-1 used Pembrolizumab and got a higher PCR rate of 55.6% (28). Meanwhile, some other trials combined chemotherapy alone with immunotherapy. Yang et al. evaluated the efficacy and safety of camrelizumab plus nab-paclitaxel and S1 capsule followed by surgery, and the PCR rate was 33.3% (29). Xing et al. designed a phase II randomized trial, in which both groups received chemotherapy on day 1, then the experimental group received toripalimab on day 3, while the control group received it on day 1. The PCR rates were 36% and 7%, respectively (30). However, none of those neoadjuvant chemoimmunotherapy studies allowed PORT, which may be due to the safety concern. The studies mentioned above are all with a small sample size, and the value of PORT for patients with EC under the brand new neoadjuvant therapeutic regimen including immunotherapy and chemotherapy needs to be redefined in the future.

However, we acknowledge several important limitations in our study. First, selection bias could not be avoided because of the retrospective nature of our study, although PSM was performed. Second, in the SEER database, it lacks detailed information regarding chemotherapy regimen, radiation dose, surgical margin, and certain risk factors such as smoking and alcohol exposure, which can affect the reliability of our findings.

In summary, our results detect no survival benefit with the use of PORT after NAC and surgery in patients with EC. Furthermore, multivariate analysis indicates that PORT, age ≥71 years old, cT3–4, and cN2–3 are independent predictors of worse OS. Further study is needed to identify an optimal treatment strategy in patients with EC after NAC and surgery.

## Data availability statement

The original contributions presented in the study are included in the article. Further inquiries can be directed to the corresponding authors.

## Ethics statement

Ethical review and approval was not required for the study on human participants in accordance with the local legislation and institutional requirements. Written informed consent for participation was not required for this study in accordance with the national legislation and the institutional requirements.

## Author contributions

S-YZ and W-XQ contributed to the design of the research, to the analysis of the data, and to the writing of the manuscript. S-GZ and J-YC were in charge of overall direction. All authors contributed to the article and approved the submitted version.

## Funding

This study was supported in part by the Clinical Research Plan of SHDC (grant numbers SHDC2020CR4070 and SHDC2020CR2052B) and special construction of integrated Chinese and Western medicine in general hospital (numbers ZHYY-ZXYJHZ X-2-201704 and ZHYY-ZXYJHZ X-2-201913).

## Conflict of interest

The authors declare that the research was conducted in the absence of any commercial or financial relationships that could be construed as a potential conflict of interest.

## Publisher’s note

All claims expressed in this article are solely those of the authors and do not necessarily represent those of their affiliated organizations, or those of the publisher, the editors and the reviewers. Any product that may be evaluated in this article, or claim that may be made by its manufacturer, is not guaranteed or endorsed by the publisher.

## References

[1] Sung H Ferlay J Siegel RL Laversanne M Soerjomataram I Jemal A. Global cancer statistics 2020: GLOBOCAN estimates of incidence and mortality worldwide for 36 cancers in 185 countries. CA Cancer J Clin (2021) 71(3):209–49. doi: 10.3322/caac.21660 33538338

[2] Cunningham D. Perioperative chemotherapy versus surgery alone for resectable gastroesophageal cancer. N Engl J Med (2006) 355(1):11–20. doi: 10.1056/NEJMoa055531 16822992

[3] Shapiro J van Lanschot JJB Hulshof MCCM van Hagen P van Berge Henegouwen MI Wijnhoven BPL. Neoadjuvant chemoradiotherapy plus surgery versus surgery alone for oesophageal or junctional cancer (CROSS): long-term results of a randomised controlled trial. Lancet Oncol (2015) 16(9):1090–8. doi: 10.1016/S1470-2045(15)00040-6 26254683

[4] Yang H Liu H Chen Y Zhu C Fang W Yu Z. Neoadjuvant chemoradiotherapy followed by surgery versus surgery alone for locally advanced squamous cell carcinoma of the esophagus (NEOCRTEC5010): A phase III multicenter, randomized, open-label clinical trial. JCO (2018) 36(27):2796–803. doi: 10.1200/JCO.2018.79.1483 PMC6145832 30089078

[5] Stahl M Walz MK Riera-Knorrenschild J Stuschke M Sandermann A Bitzer M. Preoperative chemotherapy versus chemoradiotherapy in locally advanced adenocarcinomas of the oesophagogastric junction (POET): Long-term results of a controlled randomised trial. Eur J Canc (2017) 81:183–90. doi: 10.1016/j.ejca.2017.04.027 28628843

[6] von Döbeln GA Klevebro F Jacobsen A-B Johannessen H-O Nielsen NH Johnsen G. Neoadjuvant chemotherapy versus neoadjuvant chemoradiotherapy for cancer of the esophagus or gastroesophageal junction: long-term results of a randomized clinical trial. Dis Esophagus (2019) 32(2). doi: 10.1093/dote/doy078/5078143 30137281

[7] Jing S Qin J Liu Q Zhai C Wu Y Cheng Y. Comparison of neoadjuvant chemoradiotherapy and neoadjuvant chemotherapy for esophageal cancer: a meta-analysis. Future Oncol (2019) 15(20):2413–22. doi: 10.2217/fon-2019-0024 31269806

[8] Fan M Lin Y Pan J Yan W Dai L Shen L. Survival after neoadjuvant chemotherapy versus neoadjuvant chemoradiotherapy for resectable esophageal carcinoma: A meta-analysis. Thorac Canc (2016) 7(2):173–81. doi: 10.1111/1759-7714.12299 PMC4773296 27042219

[9] Tougeron D Hamidou H Scotté M Di Fiore F Antonietti M Paillot B. Esophageal cancer in the elderly: an analysis of the factors associated with treatment decisions and outcomes. BMC Canc (2010) 10(1):510. doi: 10.1186/1471-2407-10-510 PMC2955041 20868479

[10] Xiao ZF Yang ZY Liang J Miao YJ Wang M Yin WB. Value of radiotherapy after radical surgery for esophageal carcinoma: a report of 495 patients. Ann Thorac Surg (2003) 75(2):331–6. doi: 10.1016/S0003-4975(02)04401-6 12607634

[11] Zieren HU Müller JM Jacobi CA Pichlmaier H Müller R-P Staar S. Adjuvant postoperative radiation therapy after curative resection of squamous cell carcinoma of the thoracic esophagus: A prospective randomized study. World J Surg (1995) 19(3):444–9. doi: 10.1007/BF00299187 7639004

[12] Liu T Liu W Zhang H Ren C Chen J Dang J. The role of postoperative radiotherapy for radically resected esophageal squamous cell carcinoma: a systemic review and meta-analysis. J Thorac Dis (2018) 10(7):4403–12. doi: 10.21037/jtd.2018.06.65 PMC6105941 30174889

[13] Lin H-N Chen L-Q Shang Q-X Yuan Y Yang Y-S. A meta-analysis on surgery with or without postoperative radiotherapy to treat squamous cell esophageal carcinoma. Int J Surg (2020) 80:184–91. doi: 10.1016/j.ijsu.2020.06.046 32659390

[14] Ajani JA Barthel JS Bentrem DJ D’Amico TA Das P Denlinger CS. Esophageal and esophagogastric junction cancers. J Natl Compr Canc Netw (2011) 9(8):830–87. doi: 10.6004/jnccn.2011.0072 21900218

[15] Chen S Weng H Wang G Liu D Li H Zhang H. The impact of adjuvant radiotherapy on radically resected T3 esophageal squamous cell carcinoma. J Cancer Res Clin Oncol (2016) 142(1):277–86. doi: 10.1007/s00432-015-2041-z PMC11819054 26328915

[16] Chen J Pan J Zheng X Zhu K Li J Chen M. Number and location of positive nodes, postoperative radiotherapy, and survival after esophagectomy with three-field lymph node dissection for thoracic esophageal squamous cell carcinoma. Int J Radiat OncologyBiologyPhysics (2012) 82(1):475–82. doi: 10.1016/j.ijrobp.2010.08.037 20934269

[17] Wang X Palaskas NL Yusuf SW Abe J Lopez-Mattei J Banchs J. Incidence and onset of severe cardiac events after radiotherapy for esophageal cancer. J Thorac Oncol (2020) 15(10):1682–90. doi: 10.1016/j.jtho.2020.06.014 PMC9398884 32599073

[18] Pinder-Arabpour A Jones B Castillo R Castillo E Guerrero T Goodman K. Characterizing spatial lung function for esophageal cancer patients undergoing radiation therapy. Int J Radiat OncologyBiologyPhysics (2019) 103(3):738–46. doi: 10.1016/j.ijrobp.2018.10.024 PMC7063843 30612962

[19] Zhang Z Zhang H. Impact of neoadjuvant chemotherapy and chemoradiotherapy on postoperative cardiopulmonary complications in patients with esophageal cancer. Dis Esophagus (2017) 30(4):1–7. doi: 10.1093/dote/dox002 28375486

[20] Tepper J Krasna MJ Niedzwiecki D Hollis D Reed CE Goldberg R. Phase III trial of trimodality therapy with cisplatin, fluorouracil, radiotherapy, and surgery compared with surgery alone for esophageal cancer: CALGB 9781. JCO (2008) 26(7):1086–92. doi: 10.1200/JCO.2007.12.9593 PMC5126644 18309943

[21] Arnold M Soerjomataram I Ferlay J Forman D. Global incidence of oesophageal cancer by histological subtype in 2012. Gut (2015) 64(3):381–7. doi: 10.1136/gutjnl-2014-308124 25320104

[22] Eyck BM van Lanschot JJB Hulshof MCCM van der Wilk BJ Shapiro J van Hagen P. Ten-year outcome of neoadjuvant chemoradiotherapy plus surgery for esophageal cancer: The randomized controlled CROSS trial. JCO (2021) 39(18):1995–2004. doi: 10.1200/JCO.20.03614 33891478

[23] Ando N Kato H Igaki H Shinoda M Ozawa S Shimizu H. A randomized trial comparing postoperative adjuvant chemotherapy with cisplatin and 5-fluorouracil versus preoperative chemotherapy for localized advanced squamous cell carcinoma of the thoracic esophagus (JCOG9907). Ann Surg Oncol (2012) 19(1):68–74. doi: 10.1245/s10434-011-2049-9 21879261

[24] Kitagawa Y Uno T Oyama T Kato K Kato H Kawakubo H. Esophageal cancer practice guidelines 2017 edited by the Japan esophageal society: part 1. Esophagus (2019) 16(1):1–24. doi: 10.1007/s10388-018-0641-9 PMC6510883 30171413

[25] Sun J-M Shen L Shah MA Enzinger P Adenis A Doi T. Pembrolizumab plus chemotherapy versus chemotherapy alone for first-line treatment of advanced oesophageal cancer (KEYNOTE-590): a randomised, placebo-controlled, phase 3 study. Lancet (2021) 398(10302):759–71. doi: 10.1016/S0140-6736(21)01234-4 34454674

[26] Luo H Lu J Bai Y Mao T Wang J Fan Q. Effect of camrelizumab vs placebo added to chemotherapy on survival and progression-free survival in patients with advanced or metastatic esophageal squamous cell carcinoma: The ESCORT-1st randomized clinical trial. JAMA (2021) 326(10):916. doi: 10.1001/jama.2021.12836 34519801 PMC8441593

[27] van den Ende T de Clercq NC van Berge Henegouwen MI Gisbertz SS Geijsen ED Verhoeven RHA. Neoadjuvant chemoradiotherapy combined with atezolizumab for resectable esophageal adenocarcinoma: A single-arm phase II feasibility trial (PERFECT). Clin Cancer Res (2021) 27(12):3351–9. doi: 10.1158/1078-0432.CCR-20-4443 33504550

[28] Li C Zhao S Zheng Y Han Y Chen X Cheng Z. Preoperative pembrolizumab combined with chemoradiotherapy for oesophageal squamous cell carcinoma (PALACE-1). Eur J Canc (2021) 144:232–41. doi: 10.1016/j.ejca.2020.11.039 33373868

[29] Zhang X Yang G Su X Luo G Cai P Zheng Y. Neoadjuvant programmed death-1 blockade plus chemotherapy in locally advanced esophageal squamous cell carcinoma. JCO (2021) 39(15_suppl):e16076–6. doi: 10.1200/JCO.2021.39.15_suppl.e16076 PMC8421958 34532391

[30] Xing W Zhao L Zheng Y Liu B Liu X Li T. The sequence of chemotherapy and toripalimab might influence the efficacy of neoadjuvant chemoimmunotherapy in locally advanced esophageal squamous cell cancer–a phase II study. Front Immunol (2021) 12:772450. doi: 10.3389/fimmu.2021.772450 34938292 PMC8685246

